# The Microscope in Its Relation to Medicine and Cerebral Pathology*This paper was read before the Wisconsin Academy of Sciences, Arts and Letters, July 25, 1878, at Milwaukee, Wis.

**Published:** 1879-03

**Authors:** J. N. DeHart

**Affiliations:** Late Assistant Physician to the Hospital for Insane, Mendota, Wis.


### Article II.

### The Microscope in its Relation to Medicine and Cerebral Pathology.* By J. N. DeHart, m.d., late Assistant Physician to the Hospital for Insane, Mendota, Wis.

Since the discovery of the microscope, in the 17th century, much progress has been made in many departments of science, and many revelations have been made to man, which hitherto were a hidden mystery.

Hassel in 1849 first published his Microscopic Anatomy, which was revised by Dr. VanArsdale in 1851, who issued an American edition. The volume of well executed plates which accompany this work has received much praise, which it so well merited at that day. Soon after, other works on this very important and interesting subject were published in England and America.

The many important improvements that have been made in microscopical appliances enable the student and physician of the present day to ascertain many hidden facts which, only a few years since, were not known.

While there are a great variety and number of microscopes now made and offered for sale, yet those only who have studied with the microscope know the comfort and satisfaction to be derived from the use of a good one; and by this is meant not only excellence in object glasses, although these are the most essential to a good instrument, but excellence in all the details of accessory instruments, and in nice mechanical adjustment.

Carpenter, in his valuable work, says "that of the many instruments which have been applied to scientific research, there is perhaps none that have undergone such important improvements, within so brief a space of time, as the microscope, has received the last quarter of a century; and there is certainly none whose use under its improved form has been more largely or more rapidly productive of most valuable results."

---

* This paper was read before the Wisconsin Academy of Sciences, Arts and Letters, July 25, 1878, at Milwaukee, Wis.

As an optical instrument, the microscope is now at least as perfect as the telescope. In botany, zoölogy, anatomy and physiology, the student finds the microscope a very valuable and inseparable assistant.

Every microscopist, however limited may be his opportunities, has a wide range of observations presented to him in the study of the lower forms of animal life, with the strongest incentive to persevering and well directed inquiry, that the anticipation of novelty and expectation of valuable results can afford. And it is not only in the study of the minutest forms of animal life that the microscope has been found of great service, but the anatomist and physiologist, who have made the human system their especial object of study, and had been led to believe that the information obtained by their repeated scrutiny into every portion accessible to their view, was all that lay within their power, have found in this instrument of research the means of advancing further, and of gaining a much deeper insight into the mysteries of life than had ever before been thought possible.

To the German physiologist, Schwann, who, as far back as 1839, published his "Microscopical Researches into the Structure and Growth of Animals and Plants," are we indebted for the beginning of a new era in all parts of animal physiology, which comprises the vegetative life of the organized fabric. In these researches, the author had in view the study of the development of the animal tissues. He also found that although their evolution cannot be watched while in actual progess, yet their history may be traced out by the comparison of the successive stages brought to light by the microscope; and in so far as this has been accomplished, for each separate part of the organism, the structure and action of its several components, however diverse in their fully developed condition, are found to resemble each other more and more closely, the more nearly these parts are traced back to their earliest appearance.

By this retrospective study, we find that man in the beginning was an embryonic mass, composed of a congeries of cells, all apparently similar and equal to each other; tracing this farther back, it is by the aid of the microscope ascertained that all have their origin in a primordial cell, which is the first defined product of the generative act.

In following the history of the germ from its simplest and homogeneous form to that of the completed and perfected type, we have another illustration of that law of progress from the general to the special, which is certainly one of the highest principles yet attained in the science of life. By aid of the microscope we find that the same rule applies not only to man, but also to animals and plants, which have their origin in the single cell.

And by thus persevering in our researches, by the assistance of this instrument alone, there is furnished to our visual power the history of the organic germ, from the simplest form, which, being homogeneous, seems common to every kind of living being which lives, grows, and multiplies, without showing any essential advancement upon its embryonic life.

The microscope in its use and application to the science and practice of medicine, is a very valuable adjuvant. The want of a genuine knowledge concerning the origin, nature and properties of disease germs have led Drs. Beale and Maclager to give this subject their special attention, and as the result of their labors we have two quite exhaustive works on the germ theory of disease.

Dr. Beale gives in his work several well executed drawings of disease germs, as they appeared in the microscopic field, and his views in regard to them are well received, coming from one who has written so much and so ably, after very careful microscopical research, not only at home but also abroad. He says, in relation to microscopic study, "that minute investigation in connection with disease has been most unwisely discouraged, by purely scientific men on the one hand, and by those who confine themselves to their practical medical duties on the other. By the first, because they think that medical practice affords occupation enough for one man; by the last, on the ground that scientific work unfits a man for the practical duties of the profession." It has too often happened that the very few who have devoted themselves to real medical inquiry have been unfairly treated and by the very persons who should have offered them support.

The time has now arrived when the incentive to such a course should be openly condemned, as resulting from narrow, ancient prejudices, which have long survived their allotted time. Every intelligent person should do his utmost to further these branches of investigation, which, by aid of the microscope, have already exerted so great an influence upon the discovery of the wonderful changes which occur in the human body, in health and disease, and therefore upon the progress of medicine.

The manner in which disease germs enter the human system is very ably portrayed by Dr. Beale, and his illustrations of their development in the circulation as bioplasm, as drawn from views under the microscope, are beautifully colored and delineated. While there are a variety of opinions already expressed upon this subject, yet it is admitted that they may enter through many different sources. If suspended in the air, they may pass toward or into the air cells of the lungs at each inspiration. Some of the lightest particles might reach the ultimate air cells, where an exceedingly delicate membrane, easily penetrated by living particles, alone separates them from the blood. It is already known that disease germs, like the lower vegetable and animal organisms, will live for a considerable time in water.

According to Dr. Beale's views "of all media taking part in the wide diffusion of disease germs, and facilitating their introduction into many organisms, water, there is reason to believe, is the most general and perhaps with the exception of air, the most effective." Then, again, the particles of contagious bioplasm in germinal matter may enter the body through the skin. The epidermis being swollen and moist, living particles could easily insinuate themselves between the slight chinks which exist between the epithelial cells, and gradually make their way into the capillary vessels beneath. There are also instances in which disease germs gain access to the lymphatic vessels, and grow and multiply, thereby causing abscess in some lymphatic glands. The blood is sometimes infected, and the poison then becomes general, affecting the entire system.

By the investigations of Dr. Salisbury, who commenced his microscopical researches in 1849, we are shown that the blood in certain specific diseases contains spores and embryonic filaments. In 1866 he first published his researches, and in the *American Journal of Medical Sciences* for January there is a valuable paper on this subject. He describes two new algoid vegetations, which he claims are the specific cause of certain diseases.

He claims that one of them attacks especially those histological elements, the characteristic proximate organic principle of which is either gelatin, ostein or chondrin. These are connective tissues proper, bone and cartilage. It first attacks connective tissue at the point of inoculation, and then is absorbed by the lymphatics in the vicinity of the primary lesion. Afterward we have the usual sequelæ of such specific poisons, with occasionally a hard swelling of the connective tissue. The last, he claims, is due to a too rapid development of the glue tissue cells, excited by the active growth of the germs (cypta syphilitica).

These minute organisms, are only seen with a good objective, and although a $\frac{1}{5}$ of an inch objective can detect their presence, yet a power of $\frac{1}{25}$ of an inch is required to make them appear in a perfect form. Dr. S. found this organism to be algoid in character, and it existed in multitudes, and in all stages of development, from the spore to the mature filaments. At the time of writing his paper, in 1868, he had examined over 100 cases, and uniformly found this vegetation; and, what is more interesting, he discovered that the same vegetation shows itself in the blood as soon as the disease becomes constitutional. Its presence or absence in the blood is believed to be a sure guide for commencing or discontinuing treatment.

The filaments, as they occur in the blood, are more highly refractive, and have the peculiar obtusely rounded extremities in a more marked degree than in some others. These crypta he describes as being minute, transparent, highly refractive algoid filaments, which develop in living organic matter from spores.

During the past year Dr. E. Cutter, of Boston, has been engaged in very careful microscopic researches in the same field in which Dr. Salisbury has already found so much that relates to the development of disease from germs. In these investigations Dr. Cutter not only corroborates all Dr. S. has published in regard to his discoveries, but goes even farther, and makes the existence of these disease germs more evident and positive.

The three micro-photographs, which I here present for your examination were received from Dr. Cutter a few days since, and

I trust that a close examination of them will convince those who are skeptical of these facts.

The first specimen of diseased blood was examined with a $\frac{1}{5}$ in. objective (3 class Tolles, X130), and shows copper colored spores and fat.

The second specimen was submitted to a power of $\frac{1}{50}$ of an inch objective (Tolles, X 1600), and exhibit mycelial filaments also copper colored.

The third specimen was examined with an exceedingly high power, $\frac{1}{75}$ of an inch objective (Tolles), and is magnified 1,950 times, showing an enlarged white blood corpuscle containing spores that are copper colored.

This last objective is the highest power that has been used in this country, and I do not think that there are more than one or two in use in Europe. It was made expressly for Dr. Hamman, of Boston, by Mr. Tolles, and the Doctor assists Dr. Cutter in his investigations. This objective was exhibited by Dr. Cutter at the recent meeting of the American Medical Association in Buffalo, and a very interesting lecture on the morphology of blood, accompanied by a stereopticon exhibition of these views, was given by him.

The microscope has been of incalculable aid to the student of cerebral pathology, and without it the pathologist would indeed be groping in the dark.

The lesions observed in the structure of the nerve cells, and in the pia-mater, dura-mater and arachnoid, which are associated with some forms of insanity, have opened up a field in pathological research, in which Drs. J. Crichton Browne, Ferrier, Herbert Major, Hughlings Jackson, Lockhart Clark, Meynert, Westphal and others in Europe, and Drs. Jewell, Hammond, Seguin, Schmidt, S. Weir Mitchell and Spitzka in this country, have already entered, and are directing much time and patient study to these important pathological changes. The microscope enables us to observe those lesions which occur in the gray cortex of the hemispheres, and in the medulla, pons and spinal cord.

The specimens which I shall exhibit by aid of the microscope are those of chronic mania and general paralysis or progressive paresis (and ossification of the falx cerebri). The second form of insanity, which is characterized by delusions respecting great wealth and power, is accompanied by the following physical symptoms, in which there are an unequally dilated pupil, an inequality of the facial fold, want of muscular co-ordination and quivering of the tongue. The lesions observed in the gray matter of the hemisphere in this form of insanity are a general degeneration of the nerve cells.

We usually find an adhesion of the pia-mater, and a dilated condition of the vessels, although the last is also present in cases of chronic mania. The vessels in some cases are quite tortuous, but not obstructed. While the layers of the gray matter are quite distinct, the outer layer is of normal density and presents the appearance of health.

The individual nerve cells in the smaller varieties, and sometimes in the larger pyramidal cells, present characters which are to all appearances perfectly normal. There are two pathological conditions which may be observed, and are therefore quite important.

Dr. Lockhart Clarke ascribes the first condition to a loading of the cell by pigment granules, and the cell must therefore lose its normal character. Sometimes it is not much altered in size, although it is, as it were, rounded and inflated, its branches having disappeared, and nucleus gone, we find nothing but a mass of pigment granules loosely held together.

Dr. Herbert Major claims that the above description of the transformation of nerve cells is quite exceptional. The other pathological condition consists in the presence of nerve cells of immense size, located about half way in the depth of the cortical layer. They stand out like giants among the other cells, and instantly attract the attention of the observer. They are usually of pyramidal form, but frequently their contour is irregular. Their branches are quite numerous, sometimes numbering eight or ten proceeding from a single cell. The nucleus of the cell is large, but not proportional to the size of the containing body. These large eells are usually few in number, seldom exceeding ten, but are usually less, and occasionally none are found. So far, those engaged in pathological research have found them more numerous in the parietal region than in the occipital and very rarely in the frontal lobes. While they differ in appearance from those previously described, the cell wall, nucleus and branches, being all very distinct, yet as regards their size they are not excelled. The most eminent psychologists have not advanced their opinions as to the pathological significance of these lesions, but leave the matter for further research and inquiry.

In another case of general paralysis other lesions were observed, somewhat different from those just stated.

The most external layer of gray matter presents a delicacy and whiteness to a greater degree than in health, and contrasts strongly with other pathological specimens.

There is an abnormal condition of the cell, as in other cases, and its body is shrunk, the wall being closely applied to the nucleus, which is of large size and more or less round.

As the degenerative stage progresses, no cell wall can be seen, the nucleus alone being all that is left. An inflated condition of the cell also exists.

Sometimes patches of molecular degeneration are observed in the white substance, the normal nervous structure being destroyed.

These spots have been termed by Dr. Tuke and other psychologists " miliary sclerosis."

While the above lesions are not always found on making a microscopic examination of the brains of insane persons, who have died with general paralysis or progressive paresis, yet a simple atrophy of the nerve cells is most frequently met with. It may be observed also that the nuclei of the large cells are not always swollen and rounded, as is observed in some cases of general paralysis.

In another patient who died with general paralysis the spinal cord, including the pia-mater, showed the latter, as well as the connective tissue septa radiating from it, to be thickened, and near the latter a considerable number of enlarged granule cells. On microscopic examination, a symmetrical area of the " vesicular degeneration " of Leyden was found at the floor of the sulcus collateralis, medulla cervicalis. This was small in extent, but larger on the right than on the left side. The large ganglion cells of the anterior cornua appeared some atrophic, others brittle, and still others contained large pigment clumps, or a vitreous (amyloid) condition of the protoplasm. The pyramids of the medulla oblongata were atrophic, and there was considerable connective tissue hyperplasia to be seen in them, as well as in the restiform columns. Finely developed ependymous granulations covered the floor of the fourth ventricle.

On microscopic examination of the cortex of the frontal lobe, the pia-mater was found so intimately adherent that it could not be removed from the microscopic section, and was thickened. The gray substance was diminished in depth ; the nerve elements appeared considerably diminished in number, and many triangular pericellular spaces, which usually lodge a large pyramidal nerve cell, were filled with irregular masses of detritus, with here and there a free nucleus. One nerve cell was observed which took no carmine staining, and appeared vitreous. The vessels were not well filled ; their walls appeared thickened, and were more contorted than they are in normal brain, but not thrown in knots. Deiters cells in great numbers were observed near the course of the arterioles, and in one instance a clear connection could be seen between a capillary and one of these bodies. This appearance may serve as a basis for confirming the views of Magnan and Mierzejewsky (?) that vascular new formations occur in progressive paresis, cornu ammonis atrophy. This case exhibited the permanent changes so characteristic of general paralysis, and which mark the irrecoverable damage sustained by the brain in this disease.

The atrophy of the nerve elements is in strict parallelism to that obliviousness of special subjects and events characteristic of this disease, and which is the basis of their pseudo-delusions and kleptomania, for all these can be reduced to defective, in contradistinction to perverted association.

The spinal changes are similar to those observed by Westphal, and it is not definitely settled whether they represent a secondary or primary process. We observe in these lesions of the cord the cause, which results in the inability of the patient to control and regulate certain secretions.

In making a microscopic examination of the spinal cord and brain of a patient who died with chronic mania, the following pathological lesions were found. While nothing that can be termed specifically pathological was found in the cord, yet the blood vessels were markedly injected, and there were numerous free nuclei in the adventitia of the vessels. The ganglion cells were entirely normal, with the exception of a few in the most external portion of the trigonum cervicale, which contained some pigment of a granular form; nothing noteworthy was observed in the white columns. The vessels of the brain are all injected, and thickly crowded blood corpuscles can be distinguished in the lumen of the larger and smaller arterioles; the ultimate capillaries are not so completely filled. Notwithstanding this distension of the arterioles with blood, the adventitial space appears enlarged, and in one portion of the præcentral convolutions, this adventitial space presents a cystiform dilatation. Outside the vessels and on the neuroglia border of the perivascular spaces, thickly crowded lymphoid bodies were to be seen, most marked in specimens from the frontal and temporal lobes. In many instances the track of minute vessels could be traced with a low power, by the dark rows of granules lying along this track. This is especially well seen in specimens stained with hæmatoxylin. In the nerve cells themselves no charges were observed which could be safely referred to an ante-mortem condition.

In commenting on cases of chronic mania not prominently marked by dementia, either collateral or terminal, it may be remarked that palpable changes of the essential nerve elements have never been satisfactorily shown, nor need we look for such. On the contrary, purely vascular and resulting bio-chemical changes, are fully sufficient to explain all the symptoms of such a case as the history (clinical) affords, except, of course, those dependent on perverted associations which cannot yet be referred to a somatic basis. Accordingly we find distention of the lymphatic sheaths of the cerebral vessels, as residue of long past and recent cerebral hyperæmia. The nuclei lying along the perivascular border, have, in like manner, been the result of increased intervascular pressure, and have reached their present site per diapedism. The hyperæmia formula does not point to any larvated maniacal state existing at the time of death, but to the readiness with which individuals predisposed, as the present patient was, to cerebral engagements, respond to all febrile affections by a meningeal and spinal blood fluxion.

In Jewell's *Journal of Nervous and Mental Diseases* you will find a short monograph, entitled: "Contribution to the Study of Ossification of the Meninges," in which I described a case where the falx cerebri was ossified. I have made some microscopic specimens of this interesting case, and will show you true bone as the result of this ossification. Calcareous plates situated in the cerebral membranes are frequently found in epileptics and in lepto-meningitis of long standing, as well as in pachymeningitis. True ossification is rather rare, and it usually begins in the inner surface of the cranial bones, and presents itself in the shape of spiculæ of bone. In calcareous plates, bone corpuscles are never found; in spiculæ and bony tumors they are always present. The former owe their origin merely to a deposit of calcareous matter, or salts in exudative inflammatory products. The latter are the result of a true organizing action, through the medium of cells, exactly as in normal bone. Ossification in the membranes of the brain is of very rare occurrence, but calcareous degeneration of their exudative laminæ sometimes takes place, as it is known to occur even in ganglionic cells of the brain.

Erlenmeyer found the commissure of the optic nerves hardened by deposits of calcareous matter in the brain of a monomaniac, who had died with epileptiform convulsions.

Förster, in his atlas of pathological anatomy, describes calcareous cells found in the gray substance of the lumbar enlargement of the spinal cord of a boy whose lower extremities were paralyzed.

Heschl, in Schmidt's *Jahrbücher*, 1863, is the only one, so far as I am able to ascertain, who met with what he calls an ossification of cells in the brain of a patient æt. 22 years, who died melancholic; they were in the compact substance surrounding a small hæmorrhagic cavity in the central part of the right cerebral hemisphere. He used hydrochloric acid to dissolve the granulated contents, and this left the cells with a pale outline in view.

In preparing these spiculæ for microscopic examination, I selected chromic acid (0.1 to 25.0 of water), in preference to using hydrochloric (dilute) acid. The decalcification takes place rather slowly, according to the strength of the solution, and this should be changed every few days, as the acid dissolves the lime more readily when this is frequently renewed. On microscopic examination these spiculæ were found to be true bone. Some sections of the ossified portion were placed in acid (chromic) for a few days; sections were then made, and stained with carmine in glycerine. They were then mounted in glycerine (Price's English) and submitted to an examination, which revealed the presence of the laminæ, Haversian canals and lacunæ of true bone.

Beginning, as these growths do, from that membrane which acts the part of a periosteum for the internal surface of the cranium, we are inclined to attribute the production of genuine bony plates in the dura (as well as in the falciform processes) to a relapse in the direction of its previous formative activity in infantile life.

The rapid advance in science, to which much is due in the revelations of the microscope, will I trust continue, and at no distant day receive such encouragement from all members of society that the microscope will be found in every household, and thus make a microscopist in every family.

